# Multidisciplinary educational programme for caregivers of children with atopic dermatitis- in South East Norway – an observational study

**DOI:** 10.1186/s12895-020-00119-6

**Published:** 2020-12-09

**Authors:** M. Lundborg, J.-O. H. Holm, L. Sandvik, A. H. Lossius, E. M. Rehbinder, J. C. Sitek, T. L. Berents

**Affiliations:** 1 Institute of Clinical Medicine, University of Oslo, Oslo, Norway; 2 Department of Dermatology, Oslo University Hospital, Oslo, Norway; 3 Department of Biostatistics and Epidemiology, Oslo University Hospital, Oslo, Norway

**Keywords:** Atopic dermatitis, Atopic eczema, Fear of topical corticosteroids, Multidisciplinary educational programme, Topical corticosteroid phobia, Quality of life

## Abstract

**Background:**

Educational programmes for caregivers of children with atopic dermatitis (AD) are reported to reduce the severity of AD and improve quality of life (QOL). Oslo University Hospital (OUH) in Norway offers a multidisciplinary educational programme for caregivers of children with AD.

We aimed to evaluate the AD educational programme by assessing QOL of the family, the severity of the disease and caregiver’s fear of topical corticosteroid (TCS) before and after attending the programme.

**Methods:**

This was a small observational prospective cohort study including 41 caregiver-child pairs. The children (mean age 3.4 years) had doctors’ diagnosed AD with a difficult to treat eczema. The children’s caregivers were referred from physicians to attend the AD educational programme at our hospital. At inclusion and at a 3 months follow-up QOL was assessed by Dermatitis Family Impact (DFI), the eczema severity by Patient-Orientated - SCORing Atopic Dermatitis (PO-SCORAD) and caregivers fear of TCS was recorded by asking a dichotomous “yes” or “no” question: “Are you worried about using TCS on your child?”

**Results:**

Three months after caregivers attending the educational programme there was an improvement in QOL by reduced mean DFI from 9.6 (SD 6.3) to 6.8 (SD 5.4), the mean PO-SCORAD was reduced from 38.5 (SD 15.1) to 24.6 (SD13.6), the number of caregivers reporting fear of TCS use was reduced from 33/46 (72%) to 12/41 (29%). All results *p* < 0.001.

**Conclusion:**

Our study suggests beneficial effects by improving QOL of the family, the severity of the eczema and in reducing the fear of TCS when caregivers of children with difficult to treat AD attend an AD multidisciplinary educational programme. Lack of control group makes it difficult to draw definite conclusions.

**Supplementary Information:**

The online version contains supplementary material available at 10.1186/s12895-020-00119-6.

## Background

Atopic dermatitis (AD) is a common, chronic, inflammatory skin disease with high prevalence in childhood [1]. The disease is characterized by skin barrier dysfunction, inflammation and an imbalance in the skin microbiota [1]. Children with AD have an increased risk of allergen sensitization, asthma and food allergy [2]. Atopic dermatitis is characterized by intense itch that results in impaired sleep, interferes with daily life activities and is associated with an impaired quality of life (QOL) [3], as well as having a high financial impact on families and society [4].

Disease control can be achieved by avoiding exacerbating factors and by daily, time-consuming, topical treatment [5]. Treatment is directed towards the skin barrier dysfunction and the inflammation [5, 6]. The skin barrier dysfunction is treated by the use of emollients [5, 6]. Topical corticosteroids (TCS) are first-line therapy to treat the inflammation in AD [7], however low compliance due to fear of side-effects is frequently observed [8]. This phenomenon is often referred to as “TCS phobia” or just “steroid phobia”.

Structured AD educational programmes are developed to increase the caregivers’ knowledge of several aspects of AD; the pathophysiology, clinical course, exacerbating factors and treatment [9, 10]. AD educational programmes have a primary goal to reduce the eczema severity and improve QOL of the child and its family [11].

Since 2008 Oslo University hospital (OUH) offers a multidisciplinary educational programme for caregivers of children (less than twelve years of age) with difficult to treat AD, living in the south-east part of Norway.

## Methods

This is a prospective, observational cohort study conducted from April 2015 to May 2016 at the Department of Dermatology, OUH in Norway.

The aim of this study is to evaluate the effect of caregivers attending the AD educational programme at OUH by assessing the change of the family QOL, as well as the severity of the eczema before and after attending the AD educational programme. Secondary aim is to assess any change in the caregivers reported fear of TCS after attending the AD educational programme.

Informed written consent was obtained from all caregivers before inclusion. The study was approved by the Regional Committee for Medical and Health Research Ethics South East Norway (2014/2291).

### Participants

**Fig. 1:**
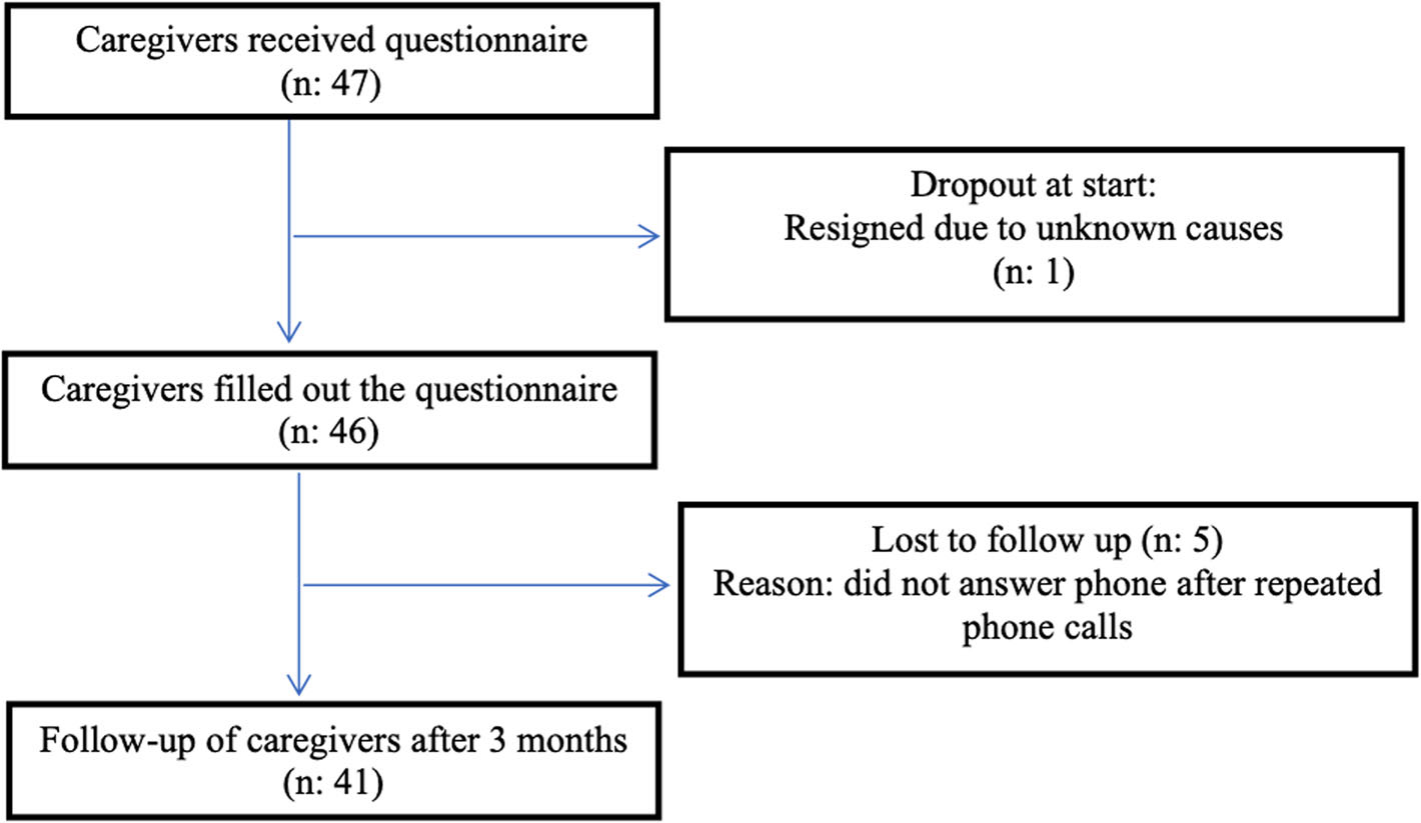
Flow chart of caregivers of children with atopic dermatitis attending a multidisciplinary atopic dermatitis training school

Caregivers of children with doctors diagnosed, difficult to treat AD referred by their physician to attend the two-day AD educational programme at OUH were invited to participate in the study. One caregiver represented one child. In total 46 caregivers of 46 children with AD were included; 21 were included from April till September, and 25 from October till March. Five participants withdrew from the follow-up (Fig. 1). We had no control group. Caregivers completed a questionnaire at inclusion and were contacted by phone for a structured interview 3 months after inclusion.

### Multidisciplinary educational programme

The multidisciplinary AD educational programme at OUH was established in 2008 after attending train-the-trainer workshop organized by the German Task Force on Educational Programmes for Atopic Dermatitis (AGNES = Arbeitsgemeinschaft Neurodermatitis Schulung) in Munich, Germany [12]. The Norwegian AD educational programme was put together in partnership with the Learning- and Health Information Centre at OUH, the Psoriasis and Eczema Association and in collaboration with an adult patient with AD.

The AD educational programme is organized four times per year. The programme is scheduled for 14 h over two-days, one week apart. Both caregivers of a maximum of 17 children are invited to each course.

The multidisciplinary educational crew consists of a dermatologist, a nurse, a psychologist, a social worker, and a representative for caregivers of children with AD and patient organisations. The structured educational programme consists of interactive group work and lectures (Supplementary Text 1). The main aim of the multidisciplinary educational programme is to educate the caregivers about AD and to give them an in-depth up-to-date overview on AD treatment. The caregivers’ attendance is provided by the social security system.

The participants are advised to strictly adhere to individual written treatment action plans prepared for the children by their physician.

### Outcomes

The QOL of the family of the child was evaluated by using the validated Norwegian version of Dermatitis Family Impact (DFI) questionnaire [13]. The questionnaire has 10 questions. Maximum score is 30.

The eczema severity score was assessed by the Norwegian version of the Patient Oriented – SCORing Atopic Dermatitis, PO-SCORAD [14]. The score represents a composite score of the signs and symptoms of the eczema. The caregiver records the extent and severity of the eczema, the areas with dry skin without eczema, and the disturbance of sleep as well as itch over the last 3 days. Maximum score is 103.

The caregivers’ fear for TCS was assessed by asking a dichotomous «yes» or «no» question: “Are you worried about using TCS on your child?”

### Assessment of outcomes

At inclusion caregivers were informed about the study and trained in performing PO-SCORAD. Paper-questionnaires including questions for DFI, PO-SCORAD and a question regarding fear of TCS use was handed out to the caregivers. One caregiver used a mobile application for PO-SCORAD. At three-month follow-up caregivers were interviewed by phone with the same questionnaire used at inclusion. The PO-SCORAD was calculated by the use of the mobile application.

### Statistical analysis

Data are presented as number and percentages, except for continuous data, which are presented as mean with standard deviation (SD) or 95% confidence intervals (CI). Paired samples t-test was used for analysing the change of continuous variables from inclusion to follow up. Mc Nemars test was used to evaluate change in fear of using topical steroids from inclusion to follow-up. Pearson correlation coefficient (r) was used to evaluate associations between variables.

Power calculation was performed prior to study start. To achieve a test power of at least 80% to detect a significant change in sum score of at least 25% when comparing inclusion and follow up data, at least 40 caregivers would have to answer the questionnaires at follow up. A drop out of about 20% was expected; therefore we aimed to include 50 patients at study start. The level of statistical significance was set to 0.05.

Statistical analyses were performed using IBM Statistical Package for Social Sciences (IBM SPSS Statistics, Version 21.0.1. Armonk, NY: IBM Corp), version 22.0.

## Results

In total 41 out of the 46 caregivers of children with AD included in this study answered questionnaires, at both inclusion and follow-up (Fig. 1). Mean age of the children included was 3.4 years (min, max; 0.4, 11.3) and 20/41 children were males, as outlined in Table 1.

**Table 1:** Descriptives of children with atopic dermatitis whose caregivers attended multidisciplinary atopic dermatitis training school at inclusion and follow-up. Mean age at inclusion was 3.4 years (min, max; 0.4, 11.3)

	Inclusion	After 3 months
Children (n)	46	41
Male sex	24 (52%)	20 (49%)
Data obtained from mother	38 (83%)	35 (85%)
Season for assessment		
April–September	21 (46%)	21 (51%)
October–March	25 (54%)	20 (49%)

A significant reduction (*p* < 0.001) of mean DFI from 9.6 (SD 6.3) to 6.8 (SD 5.4), and mean PO-SCORAD from 38.5 (SD 15.1) to 24.6 (SD 13.6) was recorded 3 months after attending the educational programme (Table 2), regardless of age and sex. At inclusion and follow-up, there was a significant correlation between PO-SCORAD and DFI; (r = 0.54, *p* < 0.001) and (r = 0.43, *p* = 0.05), respectively.

**Table 2:** Results of DFI and PO-SCORAD score at baseline and after 3 months. All values mean (SD)

	Inclusion	After 3 months)	***P***-value
DFI	9.6 (6.3)	6.8 (5.4)	< 0,001
PO-SCORAD	38.5 (15.1)	24.6 (13.6)	< 0,001

*DFI* Dermatitis Family Impact, *PO-SCORAD* Patient Oriented – Scoring Atopic Dermatitis

Fear of using TCS was reduced from inclusion to follow-up, from 33 of 46 (72%) to 12 of 41 (29%), *p* < 0,001.

## Discussion

This study shows that caregivers´ participation in our multidisciplinary AD educational programme at OUH had an improvement of family QOL and reduced eczema severity in the child. Also, there was a reduction in number of caregivers reporting fear of TCS.

Our results can indicate that caregivers attending an AD educational program is helpful in managing AD in children. This finding is in line with a Cochrane review showing some evidence that psychological and educational interventions are helpful in managing AD in children [11].

The Norwegian AD educational programme was developed after attending train-the-trainer workshop organized by the German Task Force on Educational Programmes for Atopic Dermatitis (AGNES = Arbeitsgemeinschaft Neurodermatitis Schulung), a well-studied educational programme [9, 10]. Our findings of improved family QOL and reduced eczema severity are in line with two German randomised controlled trials evaluating a multidisciplinary education programme for caregivers of children with AD, [9, 10]. One single centre study including 204 children from 5 months to 12 years with AD [9] and one multicentre study including 823 children from 3 months to 18 years of age with AD [10]. The German AD educational programme consisted of educational sessions scheduled for 2 h six afternoons each week [9, 10]. In Norway an AD educational programme in the evening would be difficult for caregivers to attend, due to long travelling route, also the social system in Norway has a well-established reimbursement practice for caregivers attending day based educational programmes. The educational programme was set to two whole days 1 week apart. The German studies showed an improvement in the QOL, using a German questionnaire, and in the severity of the eczema, using the scoring of atopic dermatitis (SCORAD). The scoring tools used in the present study were different compared to the German, therefore not directly comparable. Same scoring systems should be used so that trials can be comparable. The Harmonizing Outcome Measures for Eczema (HOME) was founded in 2008 and has developed consensus-based core outcome set for clinical AD trials; clinician-reported signs, patient-reported symptoms, quality of life and long-term control. Core outcome instruments are; Eczema Area and Severity Index (EASI), Patient Oriented Eczema Measure (POEM), The Dermatology Life Quality Index (DLQI) and the Children’s Dermatology Life Quality Index (CDLQI) and the Infants’ Dermatitis Quality of Life Index (IDQOL) and Recap of atopic eczema (RECAP) or Atopic dermatitis control test (ADCT). Future studies should include these scores [15].

The present study showed a reduction in the severity of the eczema after attending the multidisciplinary AD educational programme at OUH. Since all participants were advised to adhere to the written treatment action plan designed by their physician for their children, we postulate that the reason for the reduction in eczema severity after attending the educational programme is likely a result of better knowledge of the disease and its treatment, probably resulting in reduced anxiety for the use of TCS and possibly better treatment adherence. Better treatment adherence will give better control of the eczema and is associated with less contact with the health care system and lowers socioeconomic burden of AD [16].

The reduction in fear of TCS with targeted education reported in our study is supported by similar observations [17, 18]. A Korean study showed that fear of TCS results in low treatment adherence [18] and by reducing scepticism and avoidance of TCS by more thorough information regarding the risk of side effects and long-term use could potentially result in better treatment adherence and treatment outcome.

The strengths of our study include a prospective design with consecutive enrolment of participants throughout the year to minimalize the effect of the climate changes. The study achieved a good response rate, both at inclusion and follow-up, thus limiting the selection bias. The study is well-powered, and we have used established and well-known scoring scales [13, 14]. We chose phone interview for the follow-up investigation to avoid a lower response-rate, although this could be a limitation as the inclusion questionnaire was in paper-form.

There are several limitations of the study; low number of participants, not having a control group, and not using core outcome instruments suggested by HOME [19]. A control group is needed to show a possible effect of attending the AD educational programme. Due to lack of economic resources and time we did not have the possibility to recruit a control group for this study. Core outcome instruments for QOL was discussed at the HOME VII meeting in Japan in 2019 and recommended to be Dermatology Life Quality Index (DLQI), Children’s Dermatology Life Quality Index (CDLQI) and Infants’ Dermatitis Quality of Life Index (IDQOL). If our study had been conducted after this meeting we would have used the core outcome instruments CDLQI and IDLQI [19]. The evaluation of fear of TCS could have been evaluated in more detail by the use of a visual analogue scale or other validated scales [20]. Important factors such as asthma, educational achievement was not reported in this study. Information on this would have been interesting to adjust for in the analysis.

Future studies should include a control group to measure efficacy, have a longer follow-up period (preferably after 6 and 12 months or even longer), use core outcome instruments proposed by HOME and validated scales to measure fear of TCS.

## Conclusion

Lack of control group makes it difficult to draw conclusions. The study indicates that 3 months after caregivers of children with difficult to treat AD attended an AD educational programme the QOL of the family improved, the child had reduced severity of the eczema and there was a reduction of caregivers’ fear of TCS. Our findings support that caregiver AD educational programmes are important in the treatment of children with AD, and should be offered to caregivers of children with difficult to treat AD.

## Supplementary Information

**Additional file 1.**

## Data Availability

The data that support the findings of this study are available from the first author (JØH). Restrictions apply to the availability of these data, which were used under the license for the current study, and so are not publicly available.
