# Supplementary material for: Multidisciplinary educational programme for caregivers of children with atopic dermatitis- in South East Norway – an observational study
Source: BMC Dermatol. 2020 Dec 9;20:20. doi: 10.1186/s12895-020-00119-6 (PMC7727126; doi:10.1186/s12895-020-00119-6)
Supplement: Supplementary file 1 — Additional file 1. [file 12895_2020_119_MOESM1_ESM.docx]

Supplementary text 1

All participants introduced themselves at the start of the course. Expectations were discussed and the caregivers were encouraged to talk open about their concerns and to daily challenges with chronic sick children. The structured educational programme consisted of lectures and group work. The lectures were given by dermatologist, social worker, psychologist, dermatology nurse, a representative caregiver with child with atopic dermatitis (AD) and representatives of patient organisations. The participants were provided with written material including an educational booklet and hand-outs containing the main points from the given lectures through the two-day training programme.

At the end of day two, the caregivers were able to test their newly acquired knowledge through evaluation of pictures of children with different levels of severity of AD and learning about treatment recommendations.

**Programme day one**

09.30 - 10.00 Welcome, presentation and expectations from participants

10.00 - 10.45 Atopic dermatitis mechanisms- dermatologist

10.45 - 11.00 Coffee break

11.00 - 11.45 Treatment- dermatologist

11.45 - 12.30 Lunch – information from patient society: Psoriasis and Eczema Association

12.30 - 13.15 Triggers- allergy- dermatologist

13.15 - 13.30 Coffee break

13.30 - 14.00 Discussion in groups and plenary

14.00 - 15.00 Social economic issues- social worker

15.00 - 15.30 Discussion and conclusion

**Programme day two (one week after day 1)**

09.30 - 09.45 Short recap from day 1- any questions?

09.45 - 10.45 Coping strategies-psychologist.

10.45 - 11.00 Coffee break

11.00 - 11.45 Practical sessions: triggers and treatment – dermatology nurse

11.45 - 12.15 Lunch – information from patient society: Norwegian Asthma and Allergy Organization

12.15 - 13.15 Practical sessions: triggers and treatment – dermatology nurse

13.15 - 13.30 Coffee break

13.30 - 14.15 Practical session- clinical pictures - dermatologist

14.15 - 14.45 How to live with children with chronic AD? Caregiver of child with AD

14.45 - 15.15 Discussion. Evaluation of course.
